# Evaluation of the Effect of Oregano Essential Oil and Emulsifier Ratio on the Physicochemical, Mechanical, and Antioxidant Properties of Corn Starch Films Based on Gel Matrices

**DOI:** 10.3390/gels11090760

**Published:** 2025-09-21

**Authors:** Gabriela Uribe-Cruz, María Antonia Flores-Córdova, Mayra Cristina Soto-Caballero, Nora Aideé Salas-Salazar, María Janeth Rodríguez-Roque, Carlos Horacio Acosta-Muñiz, Claudia Andrea Romero-Bastida, Paul Baruk Zamudio-Flores

**Affiliations:** 1 Facultad de Ciencias Agrotecnológicas, Universidad Autónoma de Chihuahua, Extensión Cuauhtémoc, Barrio de la presa s/n, Ciudad Cuauhtémoc C.P. 31510, Chihuahua, Mexico; ugabriela414@gmail.com (G.U.-C.); masotoc@uach.mx (M.C.S.-C.); nsalas@uach.mx (N.A.S.-S.); mjrodriguez@uach.mx (M.J.R.-R.); 2 Centro de Investigación en Alimentación y Desarrollo, A.C. Subsede Cuauhtémoc, Fisiología y Tecnología de Alimentos de la Zona Templada. Avenida Río Conchos s/n, Parque Industrial, Ciudad Cuauhtémoc Apartado Postal 781 C.P. 31570, Chihuahua, Mexico; cacosta@ciad.mx; 3 Centro de Desarrollo de Productos Bióticos del IPN, km 8.5, Carretera Yautepec-Jojutla, Col. San Isidro, Yautepec C.P. 62731, Morelos, Mexico; cbastida@ipn.mx

**Keywords:** bio-based gel systems, biopolymeric gels, biodegradable packaging materials, starch-based gels, essential oil-based gels, conventional starch sources

## Abstract

In this study, the oregano essential oil (OEO) was extracted and physiochemically characterized in order to assess its effect on starch films formed from gel matrices. Ten formulations were proposed, in which the amounts of OEO and the emulsifier Tween^®^ 80 (Tween80) were varied in order to determine the OEO and Tween80 (*w*/*w*) ratio that would allow us to obtain a stable colloidal dispersion (without the physical perception of OEO) with an adequate incorporation of OEO. The effect of the inclusion of OEO on the rheological, physicochemical (color, thickness, and density), mechanical, water vapor permeability (WVP), and antioxidant properties of the starch-based gel films were evaluated. The formulations indicated that an OEO/Tween80 ratio of 0.0046/0.0010 g g^−1^ was the appropriate formulation for the formation of starch films from gel matrices with physical and mechanical properties suitable for being applied to food. This ratio could be ideal for obtaining films with greater mechanical properties and lower hydrophilicity (lower WVP) for packaging for foods that do not require high WVP levels.

## 1. Introduction

Currently, due to environmental pollution problems, materials are being sought that can replace or at least replicate them with common plastic materials obtained from petroleum in specific applications [1]. Developing such alternatives represents the primary objective that various research groups worldwide seek to achieve as a viable and environmentally friendly alternative [2]. In this sense, starch films formed from gel matrices (a carbohydrate synthesized by plants) are considered a suitable raw material for the production of degradable plastics or films because they have thermoplastic properties [3]. However, starch films have some drawbacks due to their hydrophilic nature, which can be solved by the addition of hydrophobic substances such as waxes, fats, and oils [4]. In this regard, OEO is considered to be a suitable hydrophobic substance to reduce the hydrophilic character [5]. In addition to the above, about OEO, it has been reported that it has antimicrobial and antioxidant properties, which are attributed to its major components such as thymol and carvacrol [6]. Recently, some works have been published where OEO has been incorporated into starch films; to mention a few studies, Hernández et al. [7] developed antimicrobial edible films based on cassava starch (*Manihot esculenta*) plasticized with glycerol, added with citric acid and OEO, and prepared by the “casting” method (using solvent casting technique). These researchers observed that adding citric acid increased the mechanical properties of tensile strength, water vapor permeability (WVP), and thermal resistance. At the same time, the OEO decreased the WVP, but produced irregularities in the microstructure of the biopolymer matrix. In addition to the above, several works have reported that the addition of essential oils in degradable films, without the presence of an emulsifying agent, leads to a separation between the lipid phase (the essential oil) and the hydrophilic phase (the polar constituents), which causes a discontinuous structure (with the lipid droplets embedded in the polymeric matrix) and can even cause pores or fractures [8].

Recently, starch-based biodegradable films have been explored as gel biopolymeric structures, due to their ability to form a cohesive network with viscoelastic properties that are similar to hydrogels. These materials can entrap active compounds such as essential oils within their matrix, offering functional properties for food packaging [9]. For this, the incorporation of OEO into starch matrices can therefore be considered a strategy not only for improving the film performance, but also to develop novel bio-based gel materials with antioxidant and barrier functionalities. Understanding the role of the OEO/emulsifier ratio is crucial for optimizing starch films formed from gel matrices networks.

Although adding hydrophobic substances (such as OEO) in degradable carbohydrate films does not represent a significant challenge, the specific incorporation in starch films formed from gel matrices depends mainly on the hydrophobic substance/emulsifier ratio. There, the scientific literature is not entirely clear on the favorable ratio and effect it can have on the mechanical and WVP properties of their respective films. For this reason, the objective of the study consisted primarily of extracting and characterizing OEO through physicochemical studies and determining its yield and antioxidant properties. This is in addition to evaluating the effect that the OEO/emulsifier ratio may have on the rheological properties of starch films formed from gel matrices solutions, and subsequently, once the films have been obtained, selecting the most suitable OEO/emulsifier ratio to be incorporated into the gel-based starch films formulation, and determining its effect on the physicochemical, mechanical, WVP, and antioxidant properties of starch films formed from gel matrices for packaging applications.

## 2. Results and Discussion

### 2.1. Yield and Physicochemical Characterization of OEO

#### 2.1.1. Yield

Table 1 shows the yield of OEO (obtained by steam distillation from 75 g of plant material), with a result of 2.20%. This value is similar to that reported by Téllez and Nolazco [10], who obtained a 2.02% yield from oregano (*Origanum vulgare* spp.) using a similar extraction method. It is also important to note that the harvest season can play a significant role in the yield obtained from the same plant, even if it is from a different species. For example, Cáceres et al. [11] reported a yield of 0.33% during the winter season, whereas this value increased to 1.1% when the OEO was extracted from *Origanum vulgare* L. harvested in spring, using steam distillation. In another recent study, Mera et al. [12] reported a lower yield (1.2 mL) than in our study, using hydro-distillation with a Clevenger apparatus from 1 kg of material. However, it has been pointed out that factors such as climatic conditions, geographic location, soil type, plant part used, and extraction method influence the final yield percentage [13]. The recent literature indicates that steam distillation offers higher yields [14].

#### 2.1.2. Density

Density is a physicochemical parameter that is important to determine for assessing the quality, purity, and authenticity of the oil [15]. According to our results, the density of OEO was approximately 0.900 g mL^−1^ (Table 1), which is a value similar to that reported by Arcila-Lozano et al. [16], who found 0.890 g mL^−1^. However, more recent studies have reported slightly higher values. For instance, Cardona and Diaz [17] reported a density of 0.942 g mL^−1^, and Téllez and Nolazco [10] reported 0.913 g mL^−1^ for OEO samples. These latter authors argue that density is also closely related to phenolic content. Therefore, we consider that phenolic content should be considered when assessing the qualitative implications of OEO density.

### 2.2. Quantification of Phenolic Compounds and Flavonoids

The quantities of phenolic compounds and flavonoids in the OEO are shown in Table 1. As observed, the concentration of phenolic compounds was 12.01 mg g^−1^, while the flavonoid content was 0.875 mg g^−1^. Therefore, it can be stated that our OEO sample contains significantly higher levels of phenolic compounds and flavonoids compared to what has been reported for substances that are considered antioxidant agents [18]. These values are consistent with the previous scientific literature regarding these types of compounds. For example, Sarfaraz et al. [19] reported a phenolic content of 31.74 mg g^−1^ and 1.89 mg g^−1^ mEqGA mL^−1^ in OEO from *Origanum vulgare*. Recently, [20] reported a phenolic content of 1.940 mEqGA mL^−1^ and 228 mEqCA mL^−1^ for flavonoids. Jung et al. [21] noted that flavonoids and other phenolic compounds can increase in response to plant stress. Some authors suggest that plants under water stress signal for stomatal closure in the leaves, activating the synthesis of phenolic compounds—especially flavonoids—to prevent cellular damage [22].

### 2.3. Antioxidant Activity Evaluated by DPPH, ABTS, and FRAP

Various methods are currently available to evaluate the OEO’s capacity to donate or accept electrons/hydrogen ions or to scavenge them (a process known as metal chelation) within its aromatic structure [23]. Antioxidant activity results are presented in Table 1. These results obtained values of 6.07 μM TE g^−1^ for DPPH, 6.64 μM TE g^−1^ for ABTS, and 225.41 μM TE g^−1^ for FRAP. The FRAP assay showed the highest antioxidant values, suggesting greater accumulation of antioxidant compounds in the OEO. It is worth noting that the first two methods detect both hydrophilic and lipophilic compounds; ABTS can quantify both types, whereas DPPH favors lipophilic compounds [24].

Baranauskaite et al. [25] assessed antioxidant activity via DPPH in EO from *Origanum vulgare*, reporting 2.605 μmol TE/100 g, which is lower than our results. Recently, Rios et al. [20] evaluated antioxidant activity in OEO using various methods, reporting values of 3.0 mM CAET mL^−1^ (DPPH), 35 mM mL^−1^ (FRAP), and 127 mM mL^−1^ (ABTS), which were also lower than in our study. Regardless of the method used, the OEO in our study showed antioxidant activity consistent with its phenolic content, which is directly linked to its antioxidant capacity [26]. Finally, according to Arango et al. [27] this capacity is attributed to major OEO components such as thymol, making density determination a key part of the oil’s physicochemical characterization.

### 2.4. Appearance of Starch Films Formed from Gel Matrices

**Figure 1:**
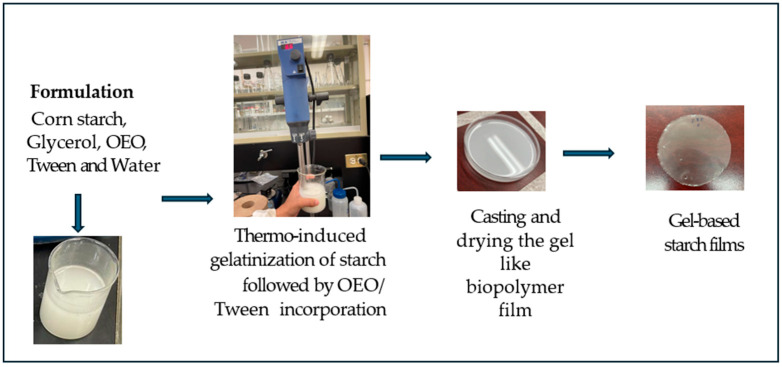
Formation and appearance of gel-based starch films based on corn starch.

Among the ten formulations tested, only the F8 formulation—comprising 4 g of corn starch, 0.0046 g of OEO, and 0.0010 g of Tween^®^ 80—resulted in a homogeneous and continuous gel-based starch film without visual separation of the lipid phase and without the presence of pores, fissures, or fractures, which is why we decided to use only this formulation. The heat induced the gelatinization process, followed by the emulsification mediated by the incorporation of the essential oil. These enabled the formation of a hydrated and cohesive polymeric network that behaved similarly to a transient hydrogel prior to drying. After solvent casting and dehydration, the resulting film exhibited uniform thickness, smooth texture, and transparency, with no visible oil droplets or fractures. These observations confirm the successful entrapment and dispersion of OEO within the starch matrix, suggesting that the selected OEO/Tween^®^ 80 ratio favors structural integrity and compatibility within the starch films formed from the gel matrices network (Figure 1).

### 2.5. Rheological Characterization of the Starch Films Formed from Gel Matrices Solutions (GFFS)

The rheological characterization of the GFFS is considered relevant because it is an important quality indicator during the production of films made from starch films formed from gel matrices as polysaccharides. A solution with high viscosity is difficult to pour, resulting in non-uniform films containing air bubbles, thus affecting their final quality [28]. Flow curves were used to obtain the rheological parameters *n* and *k* and to determine the goodness of fit (R^2^) using the Power Law model, which was found to be appropriate (R^2^ > 0.97). The results of each formulation are shown in the Supplementary Material. Therefore, these results indicated a non-Newtonian pseudoplastic behavior (also known as “shear-thinning”), as evidenced by an *n* value < 1 (Table 2).

According to several authors, pseudoplastic behavior is attributed to a slower rate of biopolymer structure reformation as the shear rate increases [29]. Pseudoplastic behavior of starch-based GFFS containing plasticizers has already been reported for corn starch films [3], as well as for native and oxidized oat starches [30], and even for chitosan-based GFFS [31]. According to the R^2^ values obtained, all formulations confirmed that the Power Law model was suitable (R^2^ > 0.97) to describe the rheological behavior, although the rheological variables did not indicate significant differences (*p* > 0.05) between the various GFFS formulations (Table 2). However, visual differences in the homogeneity of the GFFS were observed when the films were produced; in this regard, formulation 8 (referred to as F8) yielded visually complete and homogeneous films (see Table 3). Therefore, it was determined that the F8 films demonstrated the best compatibility between the hydrophobic substance (in this case, OEO) and the emulsifying agent (Tween 80), as well as with the hydrophilic phase composed of the starch biopolymer matrix, water, and plasticizer (glycerol). Thus, they were selected as the most suitable.

### 2.6. Scanning Electron Microscopy (SEM) Analysis

**Figure 2:**
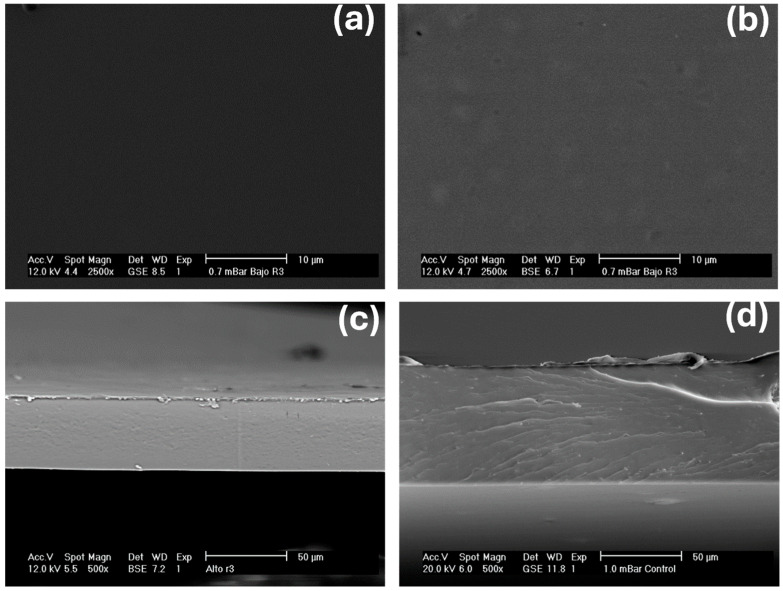
SEM micrographs of the surface (**a**) and cross-section (**c**) of the F8 film and their comparison with the surface (**b**) and cross-section (**d**) of the control film.

Figure 2 shows the surface and cross-sectional micrographs of the films of formulation F8 and their comparison with the control film. In these micrographs, it is possible to observe in the SEM images of the F8 film formulation a clear integration of the structures and the homogeneity of the OEO, which implies that in this formulation, the ratio of the emulsifier Tween^®^ 80 used was adequate for the physical incorporation of the OEO into the starch biopolymer matrix. In general terms, no significant differences were observed between these two formulations, so continuous and homogeneous surfaces were observed in both formulations, without the presence of evident fractures or irregularities, which agrees with what has been described in the scientific literature for starch matrices plasticized with glycerol, where the latter acts as a plasticizing agent and favors the formation of a compact polymeric network [32]. Regarding the cross-sectional micrographs, a greater compaction and structural integration can also be seen in the F8 film (Figure 2c) compared to the control film (Figure 2d), which indicates a greater cohesiveness in this formulation. Some recent studies have related the morphology and microstructure of biopolymeric material films (such as starch) and have even argued that they are of great importance, since they have a direct effect on other properties, such as physicochemical, mechanical and WVP properties [32,33]. Finally, we consider this result relevant in relation to some other studies that have indicated that the incorporation of OEO causes structural discontinuity in starch films at low amounts (0.1, 0.3, 0.5 and 1.0% *w*/*w* in relation to starch, when the emulsifying agent is not used), significantly impacting the physicochemical, thermal, morphological and mechanical properties of the films [33]. Therefore, we consider that the relationship between OEO and the emulsifying agent plays a predominant role in obtaining films from gel matrices with adequate incorporation, without affecting their physical and mechanical appearance.

### 2.7. Mechanical Properties Evaluation of the Films

Once the films from formulation 8 (F8) were selected, their mechanical properties (MP) were evaluated and compared with the control films (without the addition of OEO/Tween 80). The MP results showed a significant increase in tensile strength from 5.3 MPa in the control film to 7.4 MPa in the F8 film, indicating a substantial increase of approximately 40% (Table 4).

However, for the other MP variables—such as elongation at break (%E) and elastic modulus (EM)—no significant differences were observed (*p* > 0.05) between the F8 and control films. Therefore, it can be concluded that the incorporation of OEO and its interaction with the emulsifier in the F8 formulation only improved the tensile strength (TS) property. Since no significant differences were observed using SEM images (Figure 2), we hypothesize that this behavior can be explained by additional interactions between the OEO compounds and the starch matrix, possibly through secondary bonds or compatibilization effects promoted by the emulsifier, which partially reinforced the polymer network. In contrast, the properties related to elasticity and stiffness, i.e., the percentage of elongation and the elastic modulus, did not show significant increases with respect to the control film. This indicates that the addition of OEO did not modify the film’s stretchability or its elastic response to low stresses, which can be attributed to the fact that the main plasticizing phase (glycerol) continued to govern the flexibility of the system. In other words, the effect of OEO was limited to reinforcing the breaking strength, without significantly impacting the ductility or stiffness of the material. This finding can be considered positive, as it suggests that the OEO/Tween 80 ratio was appropriate for enhancing tensile strength (TS) without negatively affecting the film’s elasticity (measured by %E) or stiffness (evaluated through EM). In general, the MP values obtained in this study are consistent with recent reports from other researchers working on corn starch-based films containing chlorophyllin and hydrophobic additives, such as coconut oil, oregano essential oil, and beeswax (added at 0.5%, 1.0%, and 1.5%) [34]. However, some studies have noted that certain essential oils can disrupt polymer–polymer chain interactions, introducing a more flexible region into the film matrix and increasing %E values [35].

### 2.8. Water Vapor Permeability (WVP) Evaluation of the Films

WVP evaluation is considered essential because it indicates a film’s ability to and the rate at which it will allow water vapor molecules to pass through it, which ultimately determines its potential application in the food packaging industry [36]. According to the WVP results, the F8 film exhibited greater resistance to the passage of water molecules, indicating a lower hydrophilic character than the control film (Table 4). The WVP analysis showed that the F8 film presented lower values compared to the control film. This decrease can be explained by the hydrophobic nature of the compounds present in the AEO, which, when properly integrated into the starch biopolymer matrix due to the emulsifier Tween 80, generates areas with lower affinity for the passage of water molecules. As a result, the film structure becomes more compact and presents lower permeability, as seen in the SEM images (Figure 2).

This suggests that the F8 formulation may be suitable for use as a packaging material for products that do not require high water vapor permeability. Recent studies have reported an apparent effect of increasing OEO concentrations on improved water barrier properties, suggesting that the polyphenols present in OEO may induce a degree of hydrophobicity in the films, enhancing molecular integration within the film formulation [33]. The WVP values obtained in our study are consistent with those recently reported for starch-based films derived from cereal botanical sources. However, they can be considered low, given that some studies have reported values around 25–35 × 10^−11^ g m^−1^ s^−1^ Pa^−1^ for glycerol-plasticized corn starch films [3], while [37] reported values of 21.16 × 10^−11^ g m^−1^ s^−1^ Pa^−1^ for native potato starch films. These researchers also observed that the addition of OEO as a lipid agent reduced WVP values in proportion to the amount of OEO added, supporting the hypothesis that the water resistance of potato starch films can be synergistically improved by combining OEO addition with octenyl succinylation of the starch (a type of chemical modification). Finally, the specific ratio used in the F8 formulation may be suitable for producing corn starch films with OEO and an emulsifier at optimal concentrations, to reduce hydrophilicity (lower WVP) without needing chemical starch modification, thereby opening promising applications for the packaging of products that do not require high WVP values and help to create homogeneous materials with adequate incorporation of the AEO into the biopolymeric matrix of starch.

### 2.9. Determination of Phenolic Compounds and Flavonoids in OEO-Containing Films

In the film from formulation 8 (F8), a significant presence (*p* < 0.05) of phenolic compounds and flavonoids was detected in comparison to the control film (Table 4). According to Salgado et al. [38], this antioxidant activity is directly related to the incorporation of essential oils with high phenolic content in edible films or coatings. Some researchers even suggest that the integration of such additives stimulates biological, physical, and mechanical activities, thereby extending the product’s shelf life, improving its sensory and quality attributes, and avoiding the use of synthetic preservatives that may be harmful to health [39]. Overall, the potential outcomes of incorporating OEO into starch-based films point to possible applications in food packaging, especially for products like fresh produce or meat. In the case of meat products, some studies have explored the use of OEO as an antioxidant compound in starch-based films. For instance, Ref. [40] incorporated essential oils of oregano, clove, and rosemary into a starch film formed from gel matrices. They used a different processing method (extrusion) and added OEO at a concentration of 2%, obtaining lower phenolic compound values (1.76 μM TE g^−1^) compared to those in our study. These findings suggest that both the processing method and the type/species of plant from which the essential oil is derived may significantly influence the final phenolic and flavonoid contents in starch-based films.

### 2.10. Antioxidant Activity Evaluation (DPPH, ABTS, and FRAP) in OEO-Containing Films

The antioxidant activity of the OEO-added films was evaluated using three techniques, and significant differences (*p* < 0.05) were observed between the F8 and control films. In all cases, the F8 film exhibited higher antioxidant activity than the control, with the FRAP method showing the highest activity values. Moreover, the FRAP assay proved to be the most sensitive technique for detecting antioxidant capacity (Table 4). In a study by Cestari et al. [40], starch-based films were supplemented with OEO at 2%, resulting in an antioxidant activity of 4.06 μM TE g^−1^ using the DPPH method, lower than the values obtained in our study. Aminzare et al. [41] studied corn starch films impregnated with essential oils from *Bunium persicum* and *Zataria multiflora* and observed that the addition of OE significantly enhanced antioxidant activity, reaching up to 80% improvement over the control. In subsequent research, [42] incorporated up to 4.5% OEO into rice starch films, significantly improving mechanical strength and reducing water vapor permeability (from 8.8 to 3.7 g·mm·kPa^−1^ ·m^−2^·day^−1^, these values, in IS units similar to those reported in films, correspond from 1.02 to 4.28 g m^−1^ s^−1^ Pa^−1^, respectively), while also extending the shelf life of fish filets by reducing microbial growth and oxidation. Additionally, Wei and Shibamoto [43] reported that OEO has a preventive action by inactivating enzymes responsible for oxidation. It eliminates free radicals by donating hydrogen atoms and electrons, thus preventing the formation of oxidative substances. Due to such advantages, there has been increasing interest in natural antioxidant-active packaging compared to direct addition of antioxidants to foods [44].

## 3. Conclusions

All formulations involving different ratios of OEO to emulsifier (OEO/Tween 80) exhibited non-Newtonian pseudoplastic behavior, with no significant differences compared to the control formulation (without OEO/Tween 80). The results showed that only the formulation with an OEO/Tween 80 ratio of 0.0046/0.0010 g g^−1^ was suitable for obtaining films with the ideal physical and mechanical properties for a potential application as a packaging material. This ratio appears optimal for producing films with improved mechanical strength and reduced hydrophilic character (i.e., lower WVP), making them suitable for packaging food products that do not require high water vapor permeability. Therefore, we consider that the use of natural antioxidants and their incorporation into starch films formed from gel matrices—such as those based on starch—represents a promising field of study for the development of new materials that are safe, effective, and environmentally friendly for use in food applications.

## 4. Materials and Methods

### 4.1. Plant Material

The plant material was obtained from “El Polvo ejido” (located in the Zaragoza Valley, Chihuahua State, Mexico), whose geographic coordinates are 27°36′24.57″ N 105°40′09.89″ W at 2300 m above sea level. It is important to mention that this species of oregano (*Origanum vulgare*) had already been previously established and confirmed by botanical specialists. The sample was collected in October 2022 and transported to the plant physiology laboratory of the Faculty of Agricultural Sciences of the Autonomous University of Chihuahua (Chihuahua State, Mexico), where it was stored under laboratory conditions (HR = 65 ± 5% and temperature = 20 ± 5 °C). Therefore, the sample was dried under controlled conditions for subsequent oil extraction.

### 4.2. Extraction of OEO

OEO was obtained using the steam distillation technique according to the methodology reported by Nakas et al. [45]. Only the aerial part of the plant (leaves and flowers) was used. Seventy-five g of dried plant material was placed in a 1 L round flask with 1000 mL of distilled water, maintaining a constant extraction time of 3 h. After the distillation, the essential oil and hydrolate (aqueous phase) were separated using a 500 mL separatory funnel. The OEO was recovered and stored under refrigeration (5 °C) for the corresponding measurements.

### 4.3. Physicochemical Characterization of OEO

#### 4.3.1. Essential Oil Yield Determination

The yield (Y, defined as a %) of OEO or extract is considered the ratio (in g) of OEO obtained, depending on the quantity (in g) of dry material used, and was determined using Equation (1):

(1) $$Y\left(\%\right)=\frac{m}{m_{i}}\times 100$$

where *m* is the mass of essential oil obtained, *m*_*i*_ is the mass of plant material introduced at the beginning of the process [46].

#### 4.3.2. Determination of Essential Oil Density

Density (D) was determined at room temperature (20 ± 5 °C) using Equation (2), according to the methodology reported by Tellez Monzón and Nolazco-Cama (2017) [10].

(2) D=m/v

where

D = Density (g L^−1^)

*m* = Weight (g) of oregano (dry basis)

*v* = Volume of OEO (L)

### 4.4. Determination of Total Phenolic Content in OEO

Phenolic compounds were quantified according to the methodology proposed by Singleton et al. [47]. To do this, 1.5 mL of 2% Na_2_CO_3_ was placed in a test tube, and 0.5 mL of 50% Folin–Ciocalteau reagent was added. Subsequently, 0.3 mL of deionized H_2_O and 0.500 mL of OEO were added. The mixture was incubated at room temperature (20 ± 5 °C) in the dark for 30 min. Absorbance at 765 nm was determined using a spectrophotometer (Thermo Scientific, G 10S UV-Vis, Madison, WI, USA). Results were reported in mg of FA g^−1^ dry weight.

### 4.5. Determination of Total Flavonoid Content in OEO

Total flavonoids were quantified according to the method reported by Zhishen et al. [48]. A 250 μL extract was added, mixed with 75 μL of 5% sodium nitrite (NaNO_2_), and vortexed using an INTILLAB vortex mixer. The reaction was allowed for 5 min, then 150 μL of 10% aluminum chloride (AlCl_3_) and 500 μL of 1 M NaOH were added. The mixture was diluted to a final volume of 3 mL with distilled water. Absorbance at 510 nm was determined using a spectrophotometer (Thermo Scientific, G 10S UV Vis, USA), and flavonoids were quantified using a catechin standard curve (0.2–0.12 mg mL^−1^). The resulting values were expressed as mg catechin equivalents per g of sample (mg CE g^−1^).

### 4.6. Antioxidant Activity by the DPPH Method

Antioxidant capacity was determined using the DPPH method reported by Cardador-Martínez et al. [49]. To do this, 3.9 mg of DPPH radical was weighed and dissolved in 100 mL of 80% (*v*/*v*) methanol. The mixture was stirred constantly until a violet color was obtained. The initial reading was determined at 515 nm, and the mixture was then allowed to stand in the dark for 30 min. The results were expressed in μmol TE g^−1^.

### 4.7. Antioxidant Activity by the ABTS Method

This determination was performed according to the methodology reported by Kuskoski et al. [50]. ABTS analysis was performed by weighing 19.3 mg of ABTS and mixing it with 5 mL of distilled water. Separately, 0.0378 g of potassium persulfate was weighed in 1 mL of water. From the persulfate solution, 88 µL was taken and added to the first solution. The solution was mixed thoroughly and allowed to stand in the dark for 12–16 h at room temperature (20 ± 5 °C), until the solution turned a deep blue color. Two hundred and seventy µL of the prepared cation radical solution was taken, and 20 µL of the sample was added, reading an absorbance of 374 nm after 30 min standing time in a Thermo Scientific G 10S UV-Vis spectrophotometer (USA). The results were expressed in μmol TE g^−1^.

### 4.8. Assessment of Antioxidant Capacity Using the FRAP Method

The FRAP method is a technique that evaluates the antioxidant capacity of a substance. It is based on reducing ferric iron (Fe^3+^) to ferrous iron (Fe^2+^) in the presence of antioxidants. The antioxidant power by a reduction of the ferric ion to ferrous iron was determined using the methodology of Rubio et al. [51] with slight modifications. A stock solution was prepared under acidic conditions (pH = 3.6) and included a sodium acetate buffer solution (300 mM at a pH of 3.6). Subsequently, the iron-TPTZ complex was prepared with 20 mol of FeCl_3_·6 H_2_O in a TPTZ (2,4,6-tris(2-pyridyl)-s-triazine) solution in 40 mol of Hall’s solution. Once the stock solutions were prepared, the working solution (FRAP solution) was prepared. The solutions were added at a ratio of 10:1:1 (Buffer: FeCl_3_·6 H_2_O: TPTZ HCl). Readings were quantified at 638 nm in a Thermo Scientific G10S UV Vis spectrophotometer (USA). A standard curve was made with Trolox, with concentrations from 0 to 200 μmol. The results were expressed as μmol TE g^−1^ of dry weight of the sample.

### 4.9. Development of OEO Starch Films Formed from Gel Matrices

The film production methodologies described by Mera et al. [12] and Córsico and Larrosa [52] were used with some modifications. First, the starch films formed from gel matrices solution (GFFS) were made through thermally induced gelatinization of starch, followed by the incorporation of OEO/Tween 80 mixtures. These hydrated polymeric networks behaved similarly to transient hydrogel structures prior to drying, enabling the encapsulation and uniform distribution of hydrophobic OEO droplets. It was prepared using various formulations to vary the ratio of OEO to the emulsifier Tween^®^ 80 (the trade name for the chemical emulsifier known as polysorbate 80). A total of 10 formulations were performed; the formulations are described in Table 3, which also indicates the nomenclature used to name each of the starch films formed from gel matrices or formulation. The result of each formulation (the starch films formed from gel matrices obtained) can also be seen in Table 3. As can be seen, it was evident that only Formulation 8 (named F8) could obtain complete and homogeneous films. Consequently, it is indicated that the F8 formulation, with 4 g of corn starch, 0.0046 g of OEO, 0.0010 g of Tween 80, and the remaining amount of water for forming 100 g of total GFFS, was adequate for obtaining gel-based starch films. For this, the formulations were carried out by weighing the quantities indicated in the formulation (Table 3), subsequently stirred in the Ultra-Turrax (IKA^®^, Model T25 basic, Wilmington, NC, USA) at 6600 rpm to homogenize the components. Then, the mixture was placed on a Corning magnetic heating and stirring plate (Model PC-620D, USA), started at 24 °C, and progressively heated to 85 °C. The temperature was maintained for 5 min at a speed of 400 rpm. The starch films formed from the gel matrices solution (GFFS) were then cooled to 60 °C, and the plasticizer was added at a concentration of 2 g per 100 g of GFFS, with stirring continuing for 15 min at 400 rpm. Gel-based starch films were prepared by casting, depositing the gelatinized suspensions in polystyrene Petri dishes (P100), and drying at room temperature (20 ± 5 °C) for 48 h. The formed gel-based starch films were removed and conditioned in desiccators with a saturated NaBr solution (RH = 50 ± 5%) for 24 h. Finally, the films were stored in airtight bags (Ziploc^®^, Johnson and Sons, Inc., Racine, WI, USA) until further characterization.

### 4.10. Rheology of Starch Films Formed from Gel Matrices Solutions (GFFS)

To determine the rheological variables of the GFFS, steady-state flow curves were performed using an AR1500ex stress-controlled rheometer (TA Instruments, New Castle, DE, USA). Once the GFFS was obtained, it was allowed to cool (to 60 °C) and subjected to constant stirring (250 rpm) on a heating plate at room temperature (25 ± 5 °C). To evaluate the rheological behavior through shear tests, the methodology described by Zamudio-Flores et al. [30] was used. A 60 mm diameter stainless steel geometry with a 500 µm gap was used. Rheological tests were performed at shear rates ranging from 5 to 500 s^−1^. Flow behaviors were analyzed using the power law model: σ = k ($\dot{\gamma }$)*^n^*. Where σ is the shear stress (Pa), $\dot{\gamma }$ is the shear rate (s^−1^), *k* is the consistency index (Pa·s*^n^*), and *n* (dimensionless) is the flow behavior index. This model is preferred (compared to the Herschel–Bulkley model, for example) when yield stresses are absent, as in the case of the GFFS. The determination was carried out at least in triplicate for each of the formulations, to characterize the rheological behavior of the GFFS and determine the variables *n* and *k*.

### 4.11. Color Determination on Films

To evaluate the films’ surface color, a Minolta CR300 colorimeter (Minolta, Osaka, Japan) calibrated with a CM-A100 zero transmittance calibration tile was used. Five measurements were taken at random positions on the film using the CIELAB scale (L*, a*, b*) according to the methodology reported by Abedinia et al. [53]. The determination was performed at least in triplicate (*n* ≥ 3).

### 4.12. Evaluation of Mechanical Properties of the Films

Mechanical properties were assessed by measuring tensile strength (TS), elongation at break (%E), and elastic modulus (EM). The analysis followed the ASTM D882-95a standard and the methodology reported by Zamudio-Flores et al. [54]. Film samples were cut into rectangular strips (60 mm × 10 mm), and thickness was measured at 10 random points along each strip using a micrometer (Mitutoyo, Kobe, Japan). The average thickness was used to calculate the cross-sectional area subjected to tensile stress. Tensile tests were conducted using a TA.XTplus Texture Analyzer (Stable Micro Systems, Surrey, UK) equipped with a 30 kg load cell and a 4 cm initial grip separation, operated via Exponent Lite software (version 4.0). The test was performed at a constant extension rate of 20 mm·min^−1^. The tensile strength was calculated as the maximum force at break divided by the cross-sectional area. The elongation at break (%E) was defined as the percentage increase in length at the point of rupture (Cauchy strain). The elastic modulus (EM) was determined from the slope of the initial linear portion of the stress–strain curve. All tests were performed in triplicate (*n* ≥ 3).

### 4.13. Evaluation of Water Vapor Permeability (WVP) of Films

Water vapor permeability (WVP) was determined using the gravimetric method described in [55]. Films were cut into circular specimens (9 cm in diameter) and pre-conditioned in a desiccator at 57% relative humidity (RH), achieved using a saturated sodium bromide (NaBr) solution, at 25 ± 3 °C for 48 h. Each film was then mounted over test cells containing silica gel to generate ≈ 0% RH inside the cells. These cells were placed in a second desiccator containing a saturated sodium chloride (NaCl) solution, establishing a 75% RH environment.

The weight gain of the cells was recorded hourly for at least 7 h. The water vapor transmission rate (WVTR) was calculated using the linear regression of weight gain (Δ*w*) over time (Δ*t*), according to Equation (3):

(3) $$WVTR=\;\left(\frac{\Delta w}{\Delta t}\right)\;\times \;\frac{1}{A}$$

Subsequently, WVP was determined using Equation (4):

(4) $$WVP=\;\frac{\left(WVTR\right)\times (e)}{\Delta P}\;$$

where Δ*w* is the cell weight gain (g), Δ*t* is time (s), *A* is the exposed area of the film (0.0031 m^2^), *e* is the film thickness (m), and Δ*P* is the difference in water vapor partial pressure (Pa) between the two sides of the film. All measurements were conducted in quadruplicate at room temperature (25 ± 3 °C).

### 4.14. Extraction of Phenolic Compounds and Antioxidants from the Films

The films were stirred in ethanol (0.05 g mL^−1^) for 12 h in complete darkness to determine the phenolic compound content and antioxidant activity. After dilution, the samples were centrifuged at 16,000× *g* for 10 min at 5 °C. The supernatant was then collected, and the resulting extracts were used for the corresponding analyses.

### 4.15. Quantification of Phenolic Compounds in Films

The analyses were conducted using the same methodologies described previously, with slight modifications. For total flavonoids, the procedures established by Singleton and Ross [47] and Zhishen et al. [48] were followed. Antioxidant activity was evaluated using the ABTS assay as proposed by Kuskoski et al. [50], the DPPH radical scavenging method according to Cardador-Martínez et al. [49], and the FRAP assay based on the methodology reported by Rubio et al. [51].

### 4.16. Scanning Electron Microscopy (SEM) Evaluation of Films

The surface and cross-sectional morphology of the films were evaluated using a JEOL scanning electron microscope (JEE400, Tokyo, Japan). The film samples were adhered to the sample holder and coated with gold to make them conductive. Finally, micrographs were taken at accelerating potentials of 12 and 20 kV and a current intensity of 2 mA (images were taken at 500X and 2500X).

### 4.17. Statistical Analysis

All determinations were performed in at least triplicate (*n* ≥ 3) using a completely randomized design. The results were analyzed by one-way analysis of variance (ANOVA) using the statistical software SigmaPlot version 12.5 (Systat Software, Inc., Palo Alto, CA, USA). When significant differences were found (*p* ≤ 0.05), Tukey’s post hoc test was applied for mean comparison [56].

## Figures and Tables

**Table 1:** Physicochemical characterization, phenolic content, and antioxidant capacity of OEO *.

Analysis	Result
- Physicochemical Property	
Yield (% *w*/*w*)	2.190 ± 0.360
Density (g/cm^3^)	0.899 ± 0.008
- Antioxidant Capacity	
Phenols (mg g^−1^ GAE)	12.010 ± 0.253
Flavonoids (mg g^−1^ QE)	0.875 ± 0.013
DPPH (μmol TE g^−1^)	6.070 ± 0.012
ABTS (μmol TE g^−1^)	6.640 ± 0.016
FRAP (μmol TE g^−1^)	225.410 ± 0.010

* Mean ± standard error based on at least three replicates (*n* ≥ 3).

**Table 2:** Determination of rheological parameters and goodness-of-fit, derived from the Power Law model applied to starch films formed from gel matrices solutions (GFFS) *.

Starch Films Formed from Gel Matrices Solution Sample	Rheological Variable		
	*n*	*k*	R^2^
Control	0.528 ± 0.008 ^a^	0.841 ± 0.159 ^a^	0.997 ± 0.001 ^a^
F1	0.625 ± 0.010 ^a^	1.018 ± 0.857 ^a^	0.975 ± 0.020 ^a^
F2	0.705 ± 0.025 ^a^	1.257 ± 0.936 ^a^	0.984 ± 0.025 ^a^
F3	0.683 ± 0.033 ^a^	1.198 ± 0.877 ^a^	0.989 ± 0.010 ^a^
F4	0.711 ± 0.060 ^a^	1.308 ± 0.921 ^a^	0.995 ± 0.011 ^a^
F5	0.608 ± 0.047 ^a^	1.382 ± 0.598 ^a^	0.991 ± 0.015 ^a^
F6	0.721 ± 0.055 ^a^	1.405 ± 0.661 ^a^	0.988 ± 0.028 ^a^
F7	0.587 ± 0.039 ^a^	1.411 ± 0.705 ^a^	0.993 ± 0.033 ^a^
F8	0.477 ± 0.018 ^a^	1.466 ± 0.140 ^a^	0.995 ± 0.004 ^a^
F9	0.491 ± 0.022 ^a^	1.328 ± 0.845 ^a^	0.990 ± 0.035 ^a^
F10	0.501 ± 0.041 ^a^	1.675 ± 0.250 ^a^	0.986 ± 0.019 ^a^

* Arithmetic averages of at least three replicates (*n* ≥ 3) ± standard error. Means within each column that share the same letters are not significantly different (*p* > 0.05) according to Tukey’s test.

**Table 3:** Nomenclature, formulation, and representative image of the starch films formed from gel matrices formulations.

Film (Formulation and Nomenclature)	Formulation (To Prepare 100 g of Starch Films Formed from Gel Matrices-Forming Solution)
Formulation 1 (F1)	Starch = 4.00 g, Glycerol = 2.00 g, OEO = 0.129 g, Tween 80 = 0.0287 g, Distilled water = 93.80 g.
Formulation 2 (F2)	Starch = 4.00 g, Glycerol = 2.00 g, OEO = 0.129 g, Tween 80 = 0.0140 g, Distilled water = 93.90 g.
Formulation 3 (F3)	Starch = 4.00 g, Glycerol = 2.00 g, OEO = 0.0645 g, Tween 80 = 0.0140 g, Distilled water = 93.90 g.
Formulation 4 (F4)	Starch = 4.00 g, Glycerol = 2.00 g, OEO = 0.0323 g, Tween 80 = 0.0140 g, Distilled water = 93.90 g.
Formulation 5 (F5)	Starch = 4.00 g, Glycerol = 2.00 g, OEO = 0.1290 g, Tween 80 = 0.0140 g, Distilled water = 93.85 g.
Formulation 6 (F6)	Starch = 4.00 g, Glycerol = 2.00 g, OEO = 0.129 g, Tween 80 = 0.0140 g, Distilled water = 93.85 g.
Formulation 7 (F7)	Starch = 5.00 g, Glycerol = 2.50 g, OEO = 0.0046 g, Tween 80 = 0.0002 g, Distilled water = 92.49 g.
Formulation 8 (F8)	Starch = 4.00 g, Glycerol = 2.00 g, OEO = 0.0046 g, Tween 80 = 0.0010 g, and Distilled water = 93.99 g.
Formulation 9 (F9)	Starch = 8.00 g, Glycerol = 4.00 g, OEO = 0.800 g, Tween 80 = 0.0020 g, and Distilled water = 187.19 g.
Formulation 10 (F10)	Starch = 8.00 g, Glycerol = 4.00 g, OEO = 0.800 g, Tween 80 = 0.1738 g, and Distilled water = 187.03 g.

**Table 4:** Physicochemical properties (color, thickness, and density), mechanical properties, water vapor permeability (WVP), and antioxidant capacity of the selected film (F8) compared to the control film (without the addition of OEO) *.

Analysis	Film	
	F8	Control
- **Color Parameters**		
L	95.810 ± 0.040 ^a^	67.980 ± 9.590 ^b^
a	−0.360 ± 0.010 ^a^	−0.350 ± 0.060 ^a^
b	3.620 ± 0.040 ^a^	3.420 ± 0.440 ^a^
C	3.630 ± 0.040 ^a^	3.440 ± 0.440 ^a^
°h	95.590 ± 0.120 ^a^	69.590 ± 9.750 ^b^
- **Thickness (mm)**	0.155 ± 0.006 ^b^	0.208 ± 0.004 ^a^
- **Density (g/cm^3^)**	1.000 ± 0.002 ^a^	1.001 ± 0.010 ^a^
- **Mechanical Properties**		
TS (MPa)	7.440 ± 0.580 ^a^	5.250 ± 0.323 ^b^
%E (%)	45.470 ± 9.820 ^a^	51.380 ± 2.220 ^a^
EM (MPa)	181.600 ± 37.230 ^a^	220.330 ± 22.320 ^a^
- **WVP × 10^−11^ (g m^−1^ s^−1^ Pa^−1^)**	1.230 ± 0.001 ^b^	2.910 ± 1.340 ^a^
- **Antioxidant Capacity**		
Total Phenolic Content (mg GAE g^−1^)	2.337 ± 0.020 ^a^	0.250 ± 0.036 ^b^
Total Flavonoid Content (mg CE g^−1^)	0.025 ± 0.003 ^a^	0.027 ± 0.001 ^a^
DPPH (μmol TE g^−1^)	1.031 ± 0.067 ^a^	0.260 ± 0.012 ^b^
ABTS (μmol TE g^−1^)	1.188 ± 0.033 ^a^	0.570 ± 0.025 ^b^
FRAP (μmol TE g^−1^)	93.610 ± 3.776 ^a^	9.444 ± 0.694 ^b^

* Arithmetic averages of at least three replicates (*n* ≥ 3) ± standard error. Means values within each row and for each type of analysis followed by different letters are significantly different (*p* < 0.05), according to Student’s *t*-test post hoc analysis.

## Data Availability

No data was used for the research described in the article.

## Supplementary Materials

The following supporting information can be downloaded at: https://www.mdpi.com/article/10.3390/gels11090760/s1.
