# Supplementary material for: Evaluation of the Effect of Oregano Essential Oil and Emulsifier Ratio on the Physicochemical, Mechanical, and Antioxidant Properties of Corn Starch Films Based on Gel Matrices
Source: Gels. 2025 Sep 21;11(9):760. doi: 10.3390/gels11090760 (PMC12469715; doi:10.3390/gels11090760)
Supplement: Supplementary file 1 [file gels-11-00760-s001.zip › MEB F8 vs Control.pptx]

## Slide 1
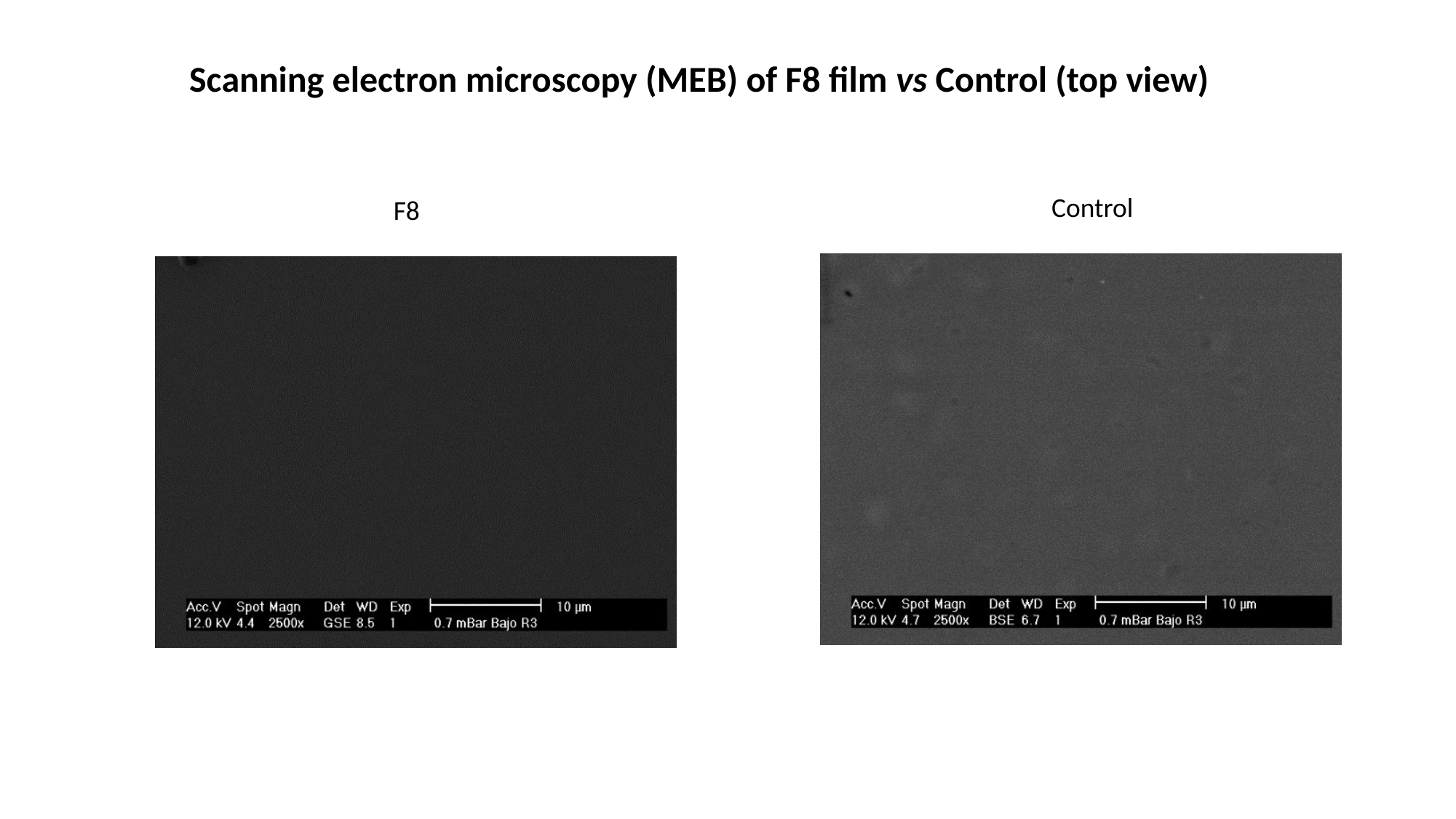

Scanning electron microscopy (MEB) of F8 film vs Control (top view)
Control
F8

## Slide 2
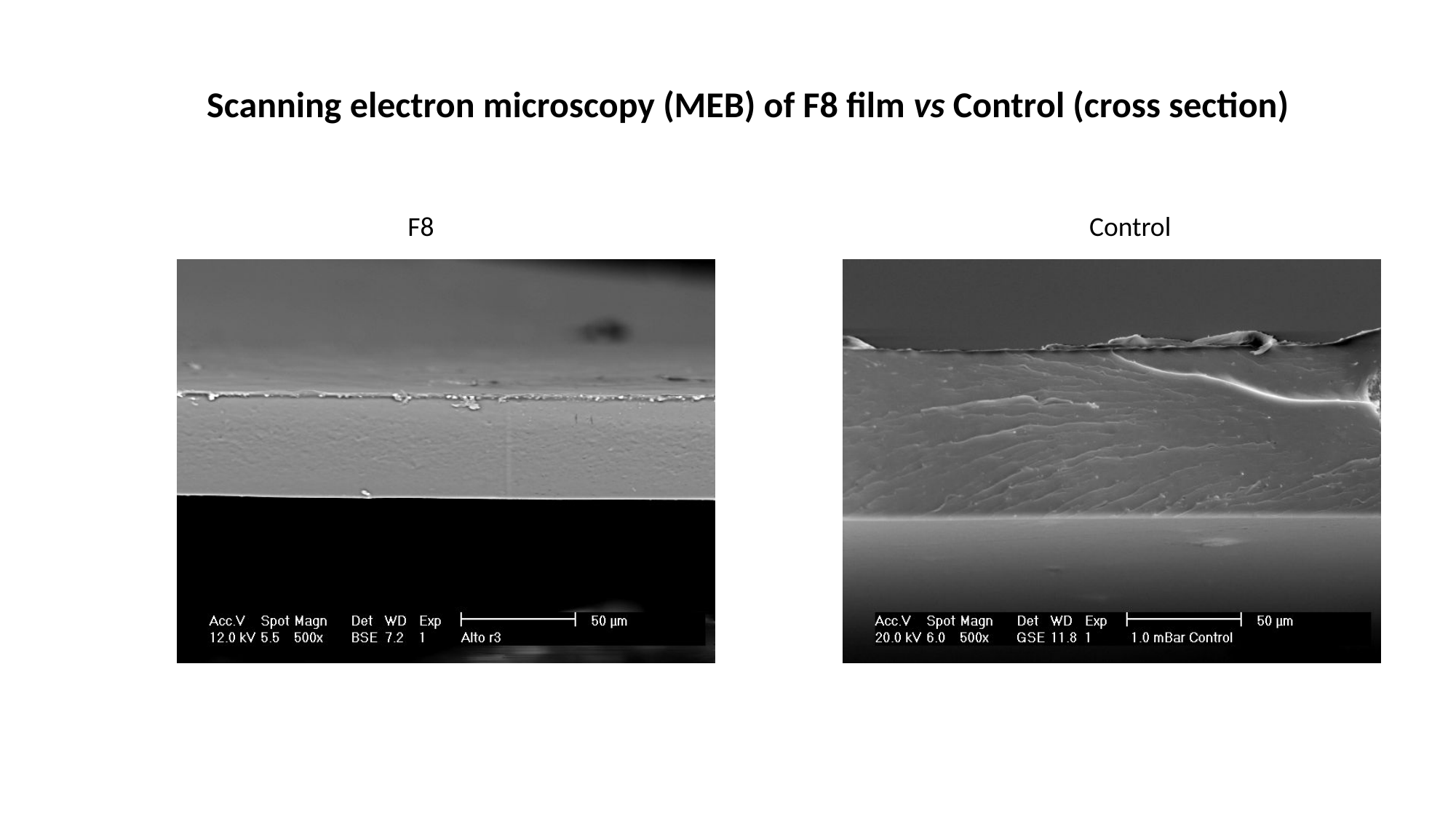

Scanning electron microscopy (MEB) of F8 film vs Control (cross section)
F8
Control
