# Supplementary figures and images for: Evaluation of the Effect of Oregano Essential Oil and Emulsifier Ratio on the Physicochemical, Mechanical, and Antioxidant Properties of Corn Starch Films Based on Gel Matrices
Source: Gels. 2025 Sep 21;11(9):760. doi: 10.3390/gels11090760 (PMC12469715; doi:10.3390/gels11090760)

Control

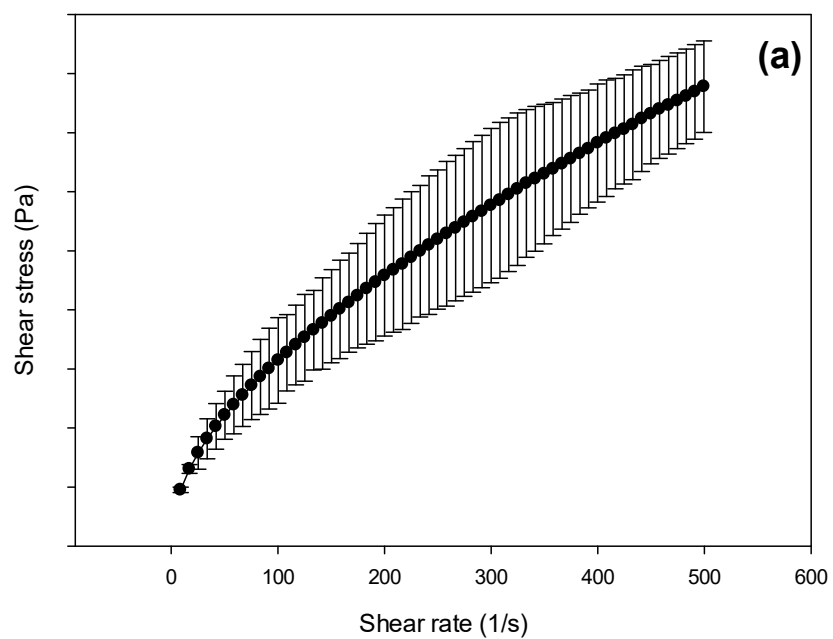

Control

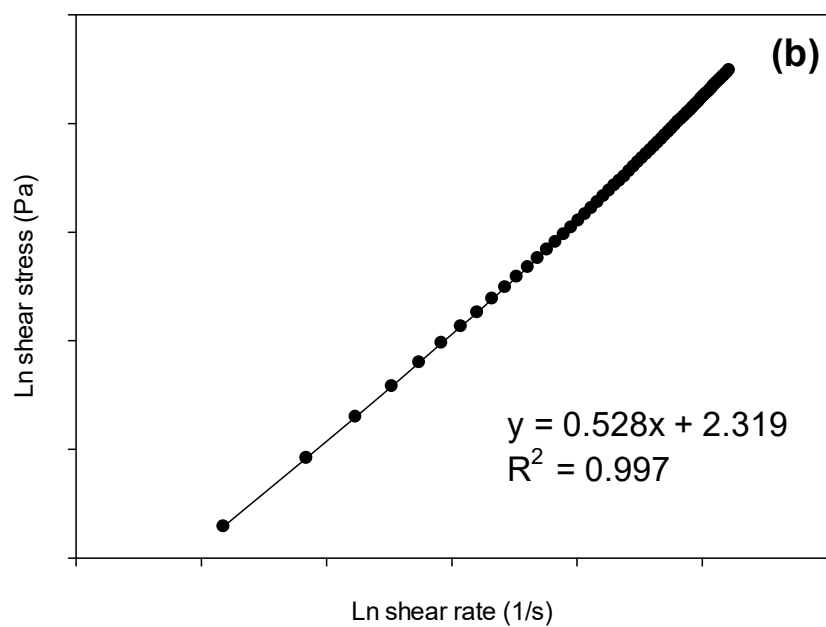

Flow curve of the Control formulation (a) and its fit to the Power Law model (b).

Supplement: Supplementary file 1 [file gels-11-00760-s001.zip › Flow curve Control.pdf]

F1

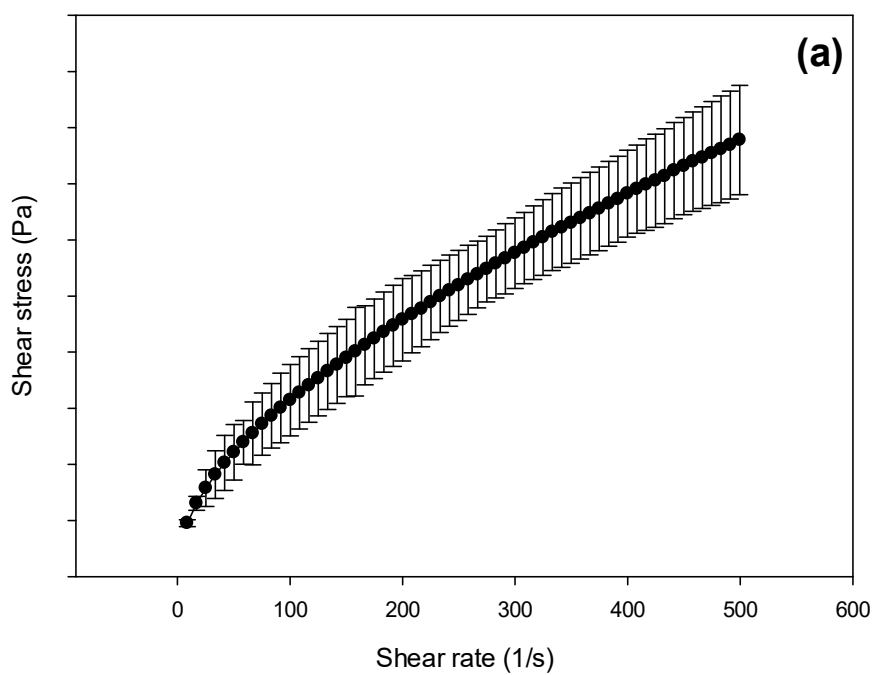

F1

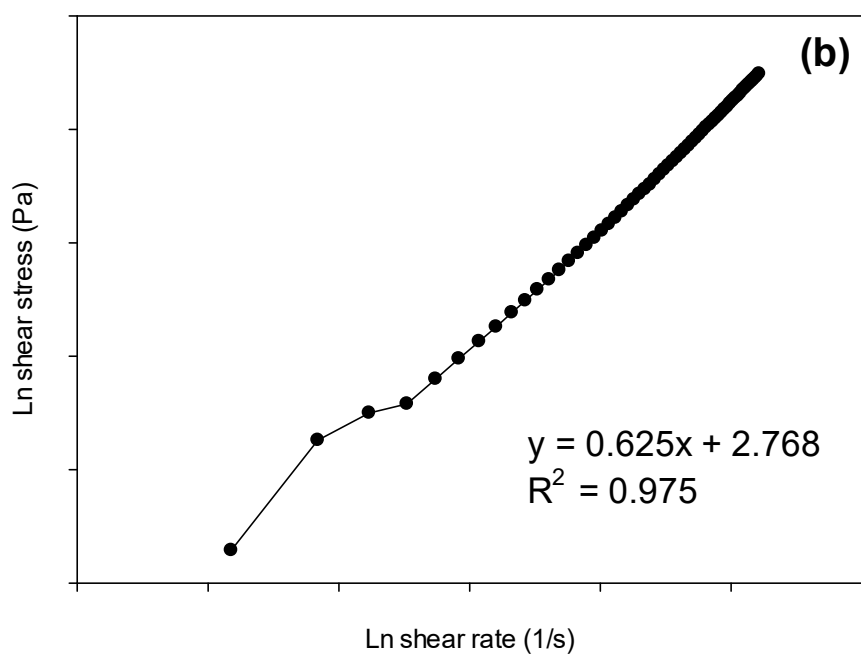

Flow curve of the F1 formulation (a) and its fit to the Power Law model (b).

Supplement: Supplementary file 1 [file gels-11-00760-s001.zip › Flow curve F1.pdf]

F10

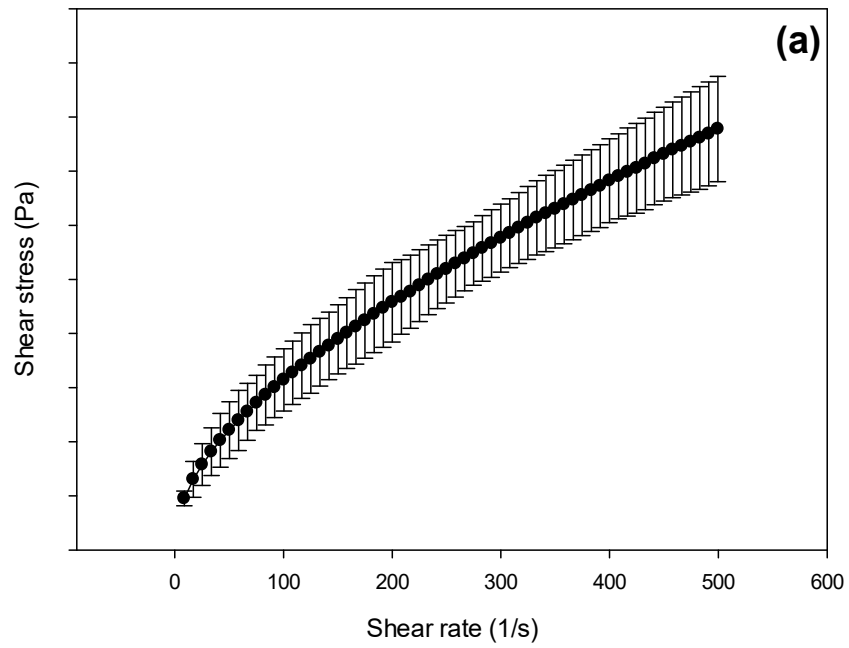

F10

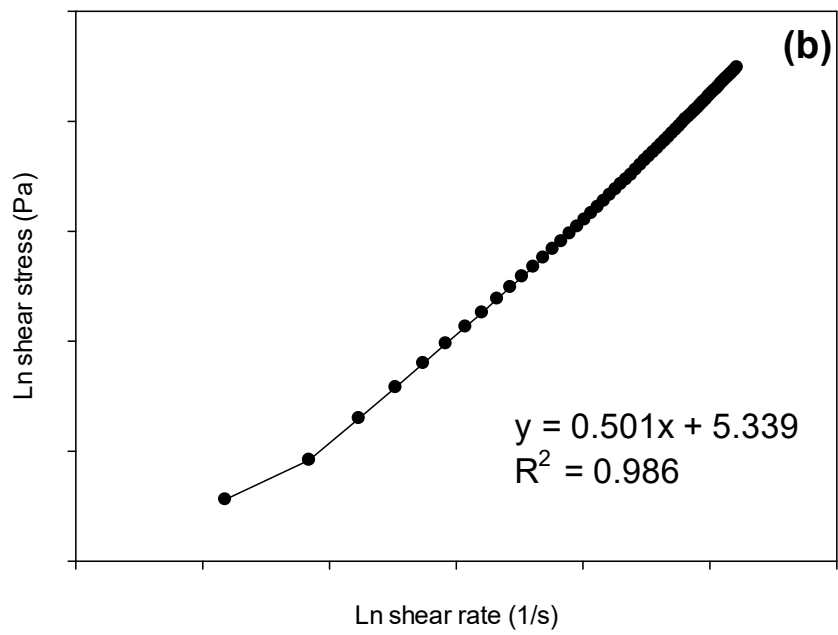

Flow curve of the F10 formulation (a) and its fit to the Power Law model (b).

Supplement: Supplementary file 1 [file gels-11-00760-s001.zip › Flow curve F10.pdf]

F2

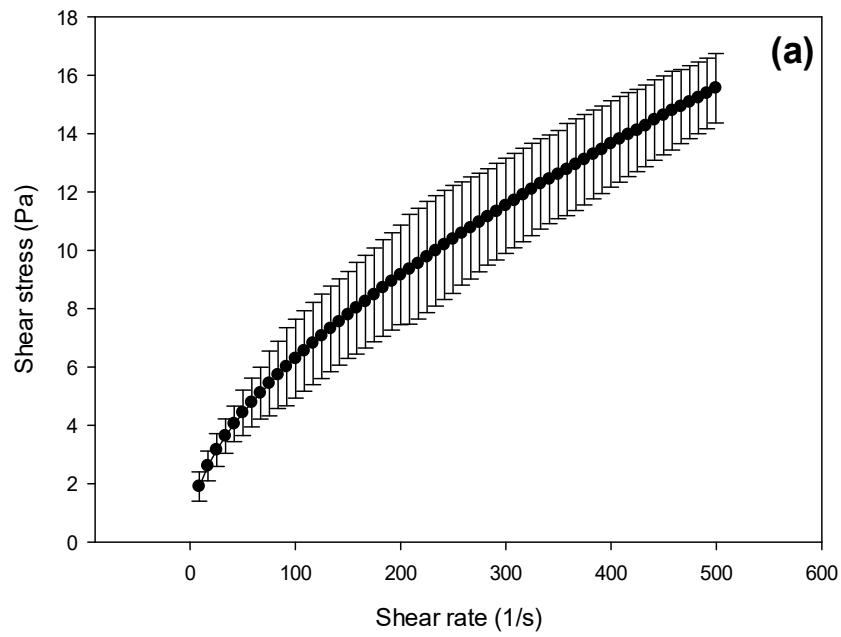

F2

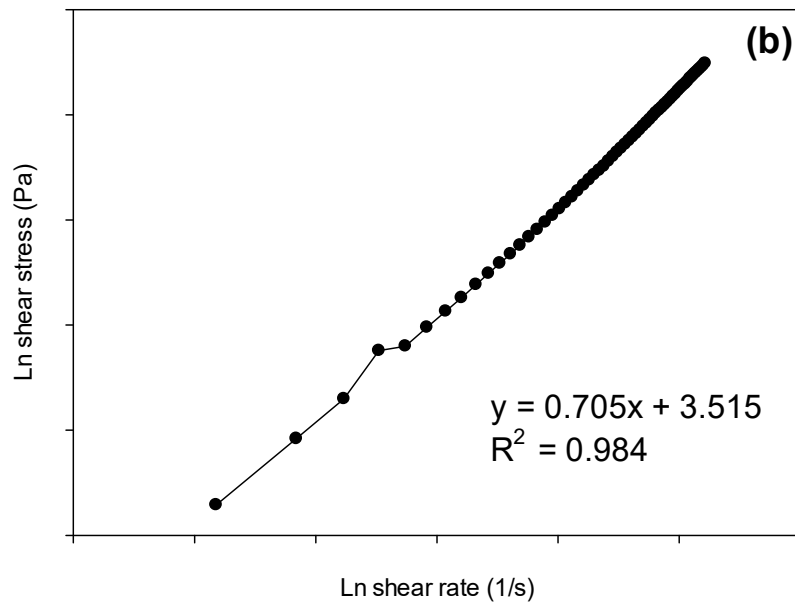

Supplement: Supplementary file 1 [file gels-11-00760-s001.zip › Flow curve F2.pdf]

F3

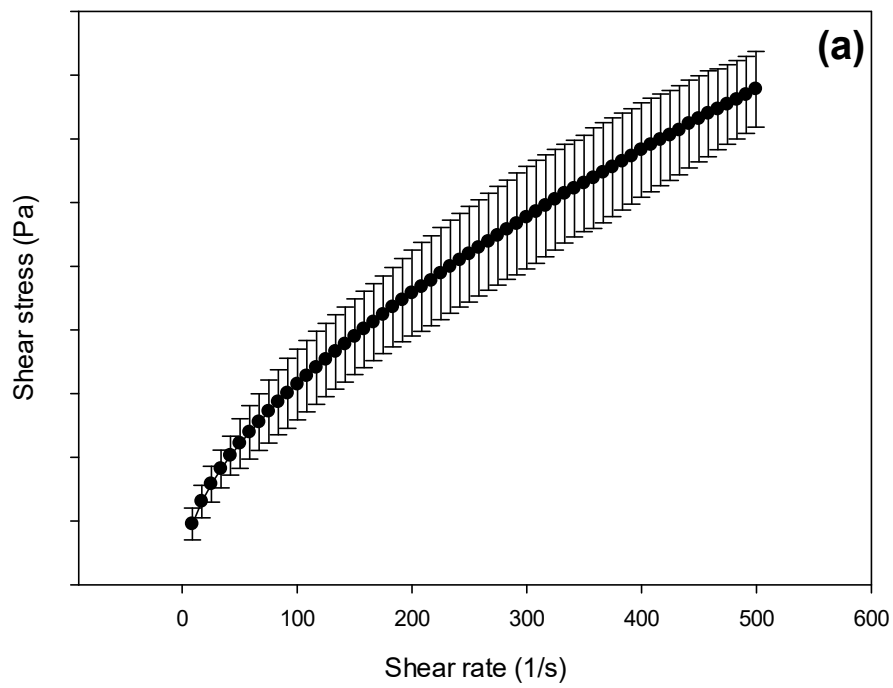

F3

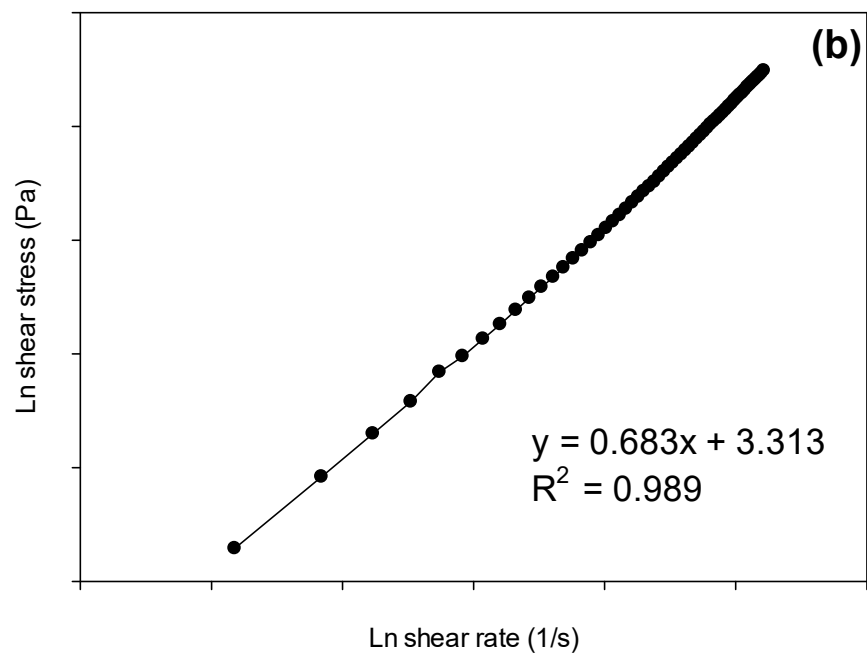

Flow curve of the F3 formulation (a) and its fit to the Power Law model (b).

Supplement: Supplementary file 1 [file gels-11-00760-s001.zip › Flow curve F3.pdf]

F4

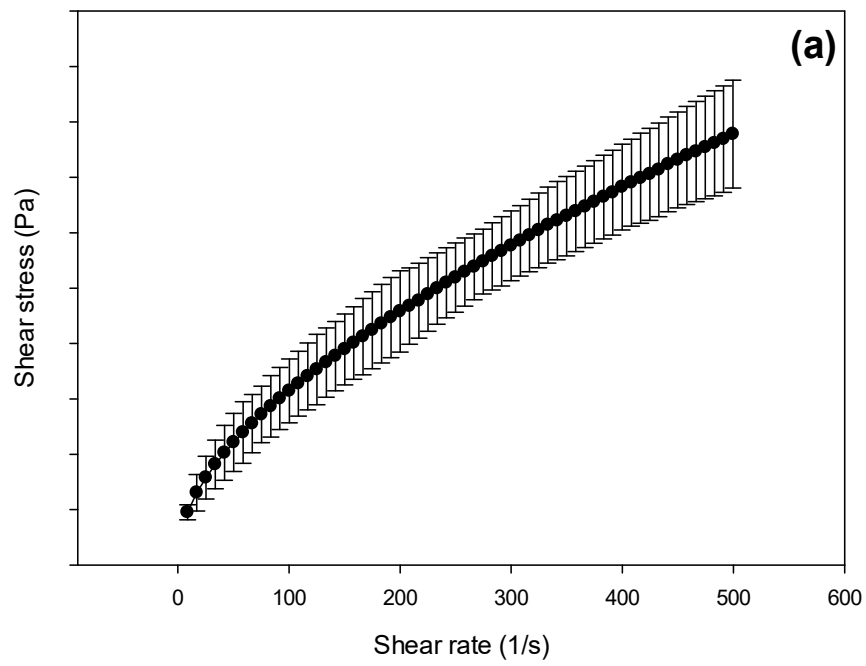

F4

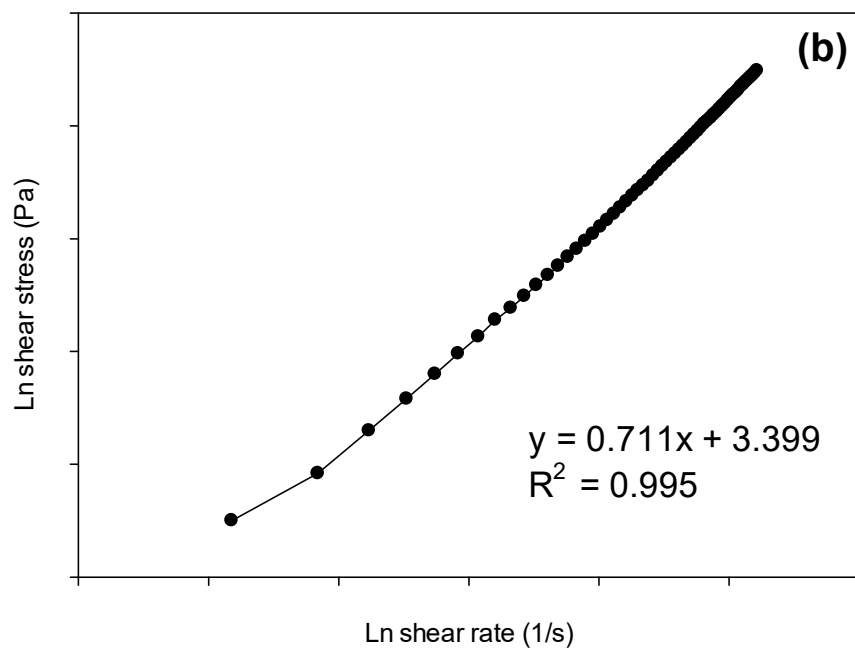

Flow curve of the F4 formulation (a) and its fit to the Power Law model (b).

Supplement: Supplementary file 1 [file gels-11-00760-s001.zip › Flow curve F4.pdf]

F5

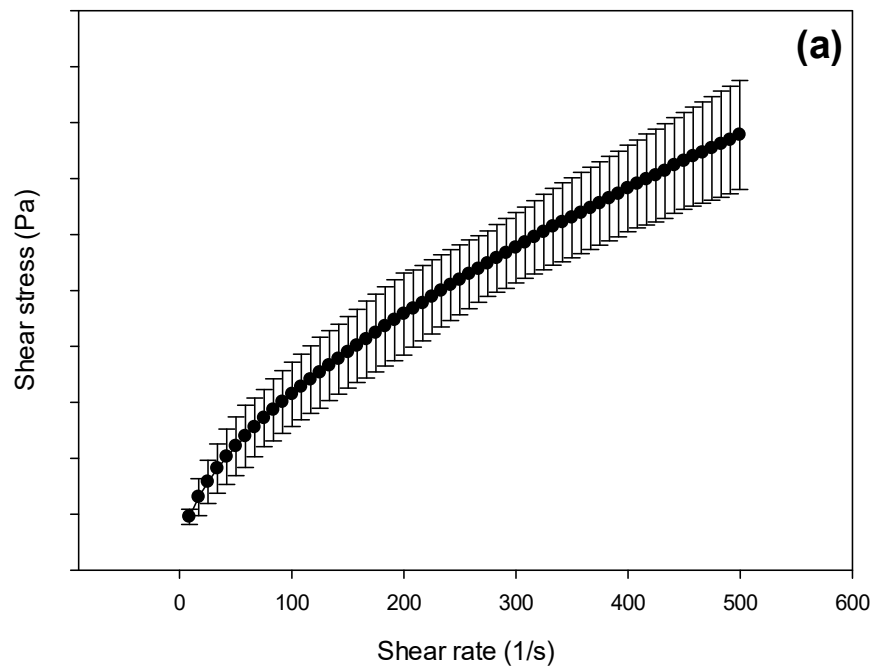

F5

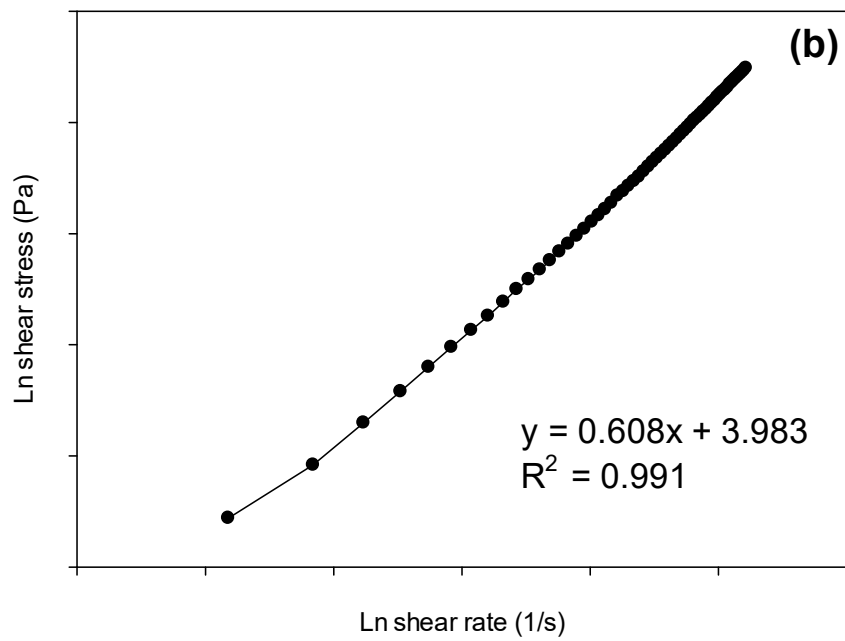

Flow curve of the F5 formulation (a) and its fit to the Power Law model (b).

Supplement: Supplementary file 1 [file gels-11-00760-s001.zip › Flow curve F5.pdf]

F6

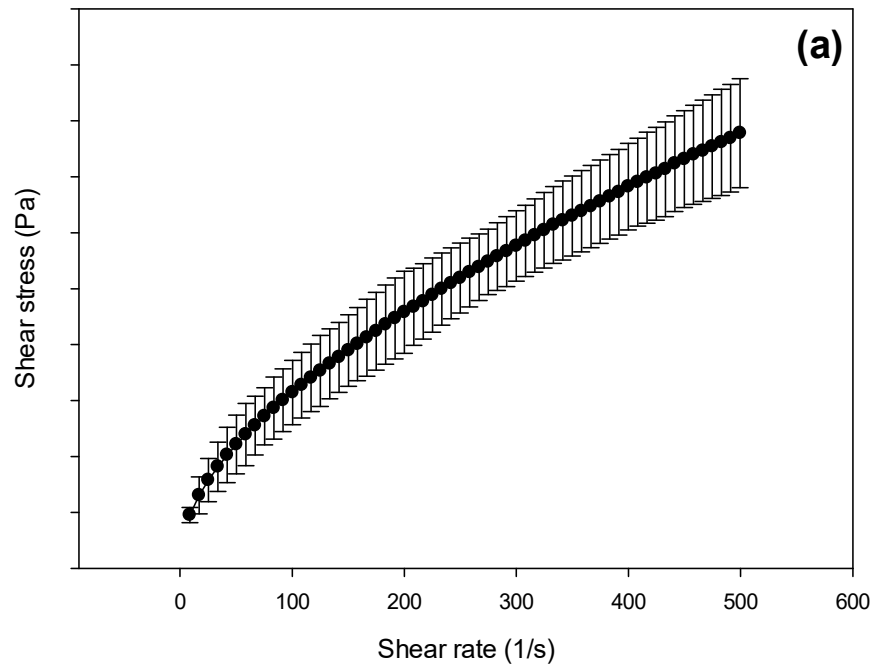

F6

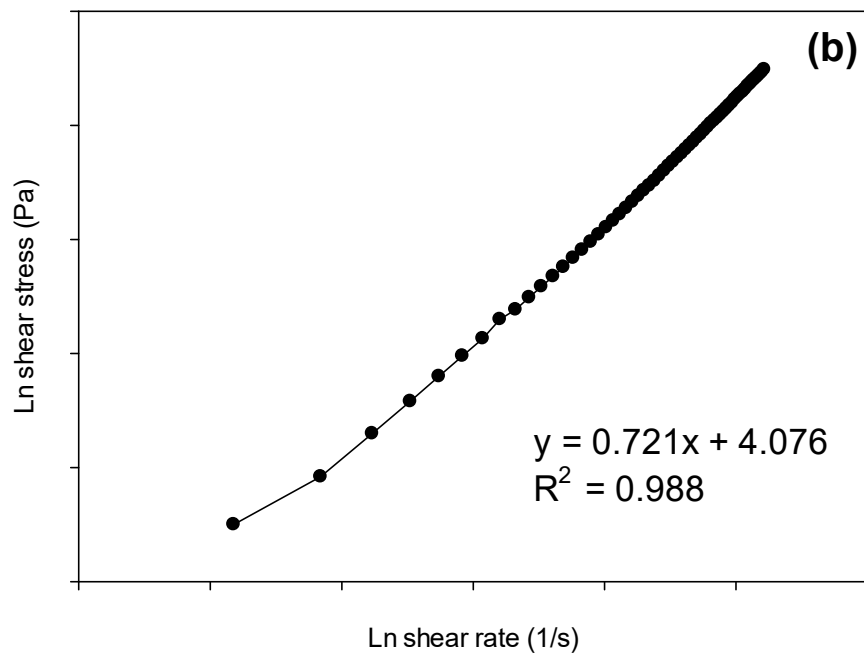

Flow curve of the F6 formulation (a) and its fit to the Power Law model (b).

Supplement: Supplementary file 1 [file gels-11-00760-s001.zip › Flow curve F6.pdf]

F7

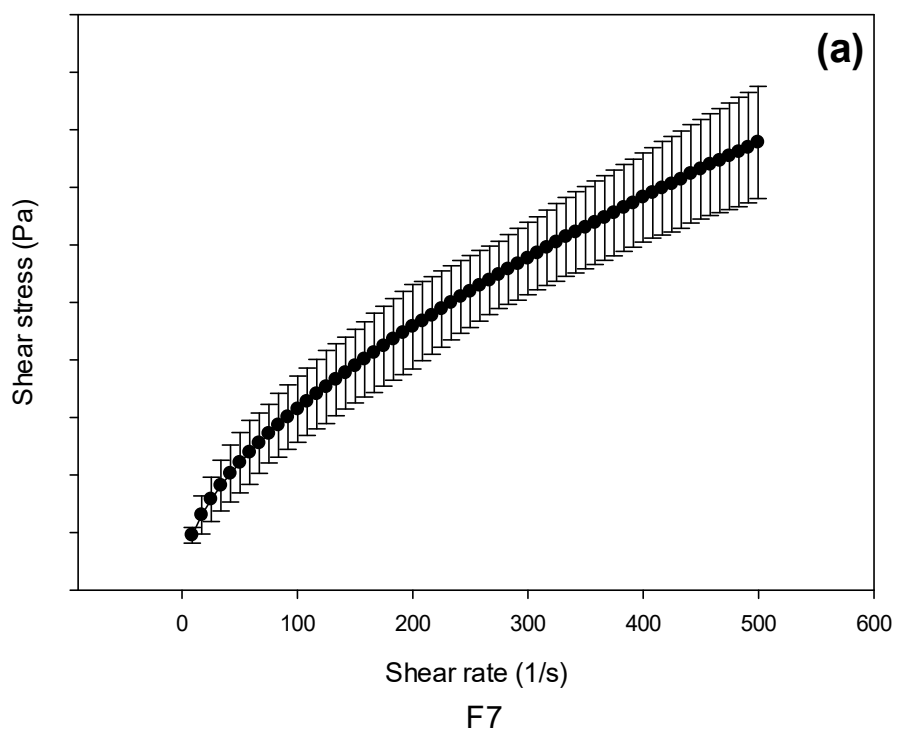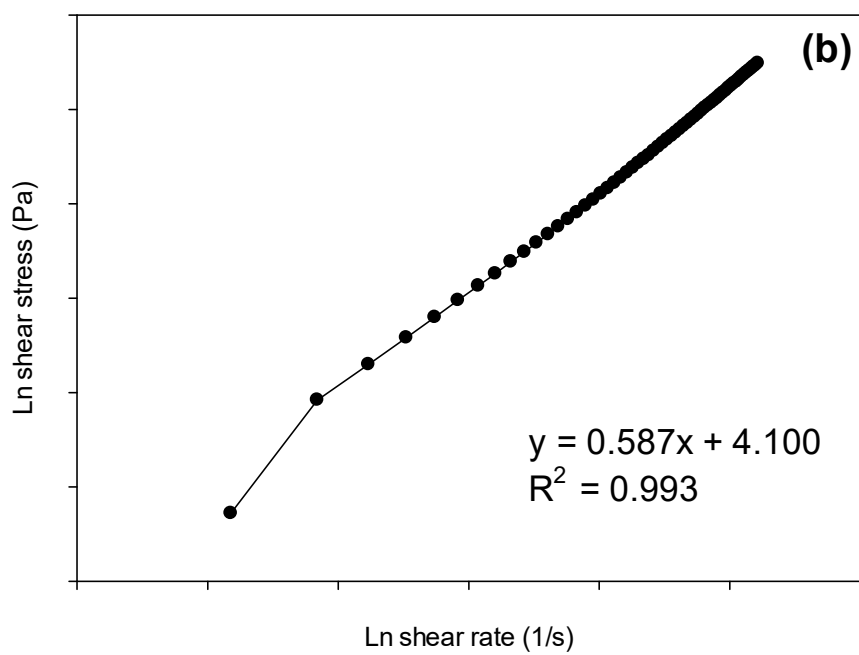

Flow curve of the F7 formulation (a) and its fit to the Power Law model (b).

Supplement: Supplementary file 1 [file gels-11-00760-s001.zip › Flow curve F7.pdf]

F8

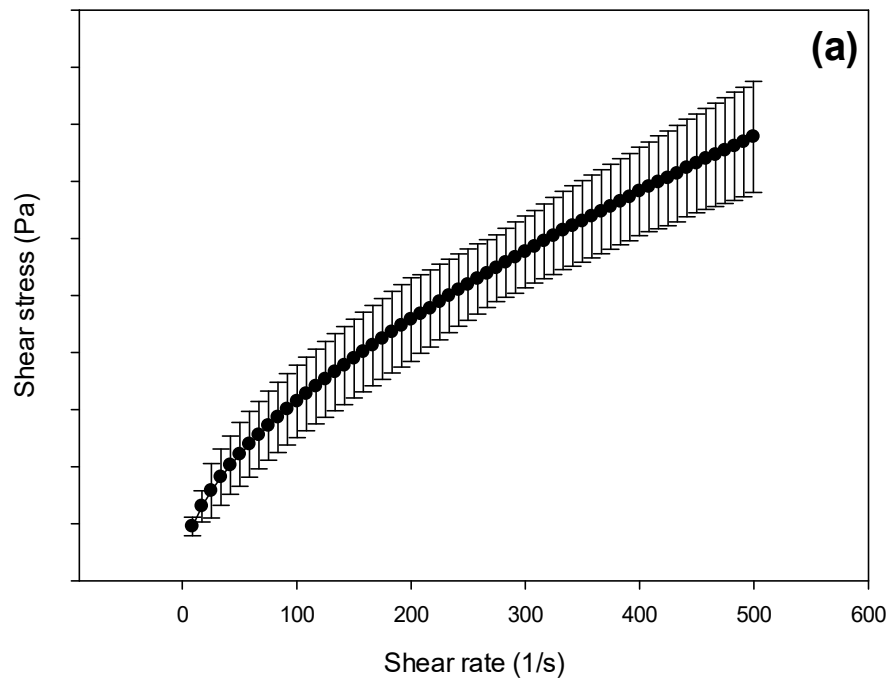

F8

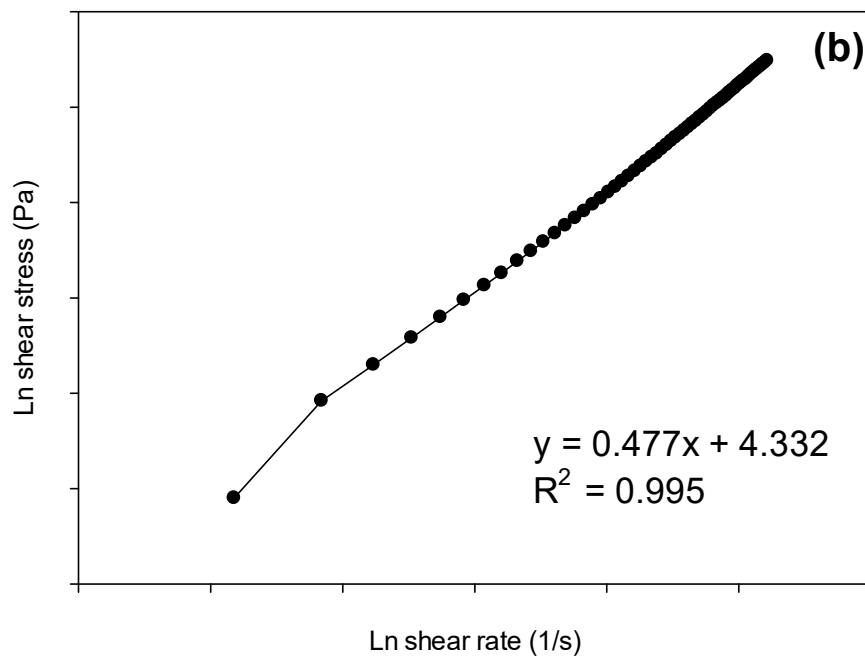

Flow curve of the F8 formulation (a) and its fit to the Power Law model (b).

Supplement: Supplementary file 1 [file gels-11-00760-s001.zip › Flow curve F8.pdf]

F9

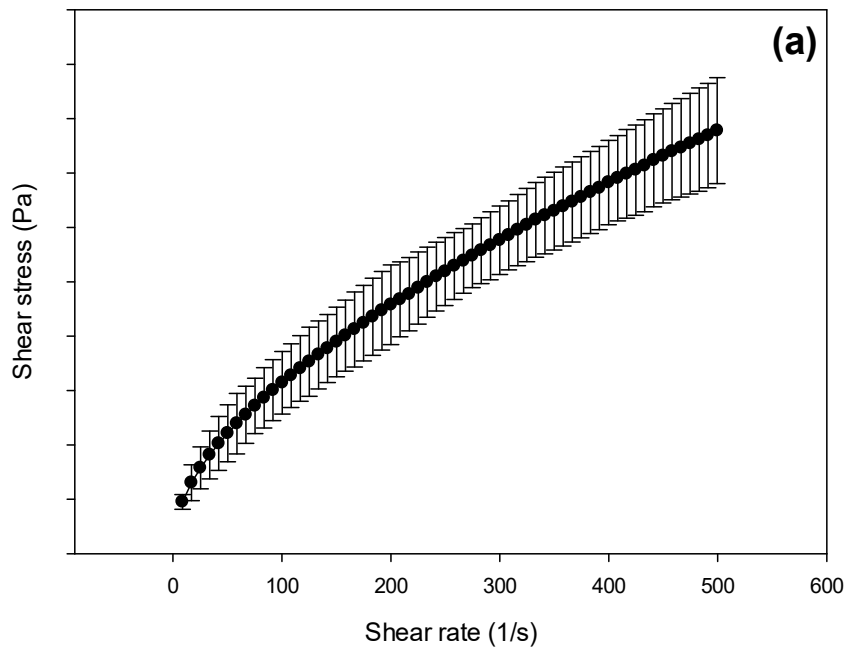

F9

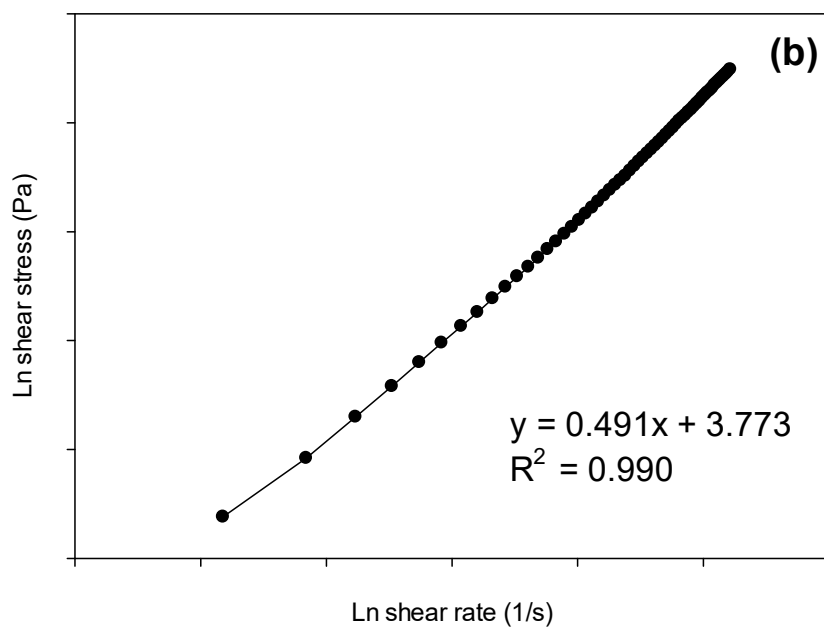

**Flow curve of the F9 formulation (a) and its fit to the Power Law model (b).**

Supplement: Supplementary file 1 [file gels-11-00760-s001.zip › Flow curve F9.pdf]
